# Internet Addiction among Students of a School in Rupandehi District, Nepal: An Observational Study

**DOI:** 10.31729/jnma.8921

**Published:** 2025-03-31

**Authors:** Bikram Kafle, Yashoda Bagale, Suraj Tiwari, Niraj Pandey, Nabin Pahari

**Affiliations:** 1 Department of Psychiatry, Devdaha Medical College, Bhaluhi, Rupandehi, Nepal; 2 Crimson College of Technology, Bhaluhi, Rupandehi, Nepal; 3 Mental Hospital, Lagankhel, Lalitpur, Nepal; 4 Department of Anatomy, Devdaha Medical College, Bhaluhi, Rupandehi, Nepal

**Keywords:** *internet addiction disorder*, *schools*, *students*

## Abstract

**Introduction:**

Students use internet for various purposes like social networking, playing online games, academic purpose, etc. Excessive Internet use has been associated with adverse psychosocial development, social isolation, impaired social skills, anger and mental health issues. This study aims to find out the prevalence of internet addiction disorder among school going adolescent of a government school of Nepal.

**Methods:**

An observational cross-section study conducted among students of Shree Shanti Model Secondary School after obtaining ethical approval from the Institutional Review Committee. Data was collected from 1 October to 1 November 2023. A convenience sampling method was used. A standard tool Young Internet Addiction Test (IAT) was used to find out if addiction was present or not.

**Results:**

Out of a total of 461 students, 137 (29.72%) had mild internet addiction, 21 (4.56%) had moderate addiction, and 303 (65.72%) had no addiction. The mean age of the students was 14.93±0.73 years. A total of 226 (49.02%) were male, and 235 (50.98%) were female. Out of total, 388 (84.16%), used mobile phones for internet access, and the main purpose of internet use was entertainment, reported by 255 (55.31%). YouTube was viewed by 266 (61.99%) users, TikTok by 77 (16.71%), and Facebook by 46 (9.98%).

**Conclusions:**

This study highlights that approximately one-third of students in a government school in Rupandehi, Nepal, exhibit mild to moderate levels of internet addiction. The findings suggest that internet use is predominantly for entertainment, with mobile phones being the most common device used.

## INTRODUCTION

Adolescents use internet for communicating, academics, and entertainment. This can lead to the internet addiction.^12^ Internet Addiction is defined as the excessive/uncontrollable use of Internet leading to symptoms of withdrawal and tolerance.^3^ Excessive Internet can lead to adverse psychosocial development and functioning (social isolation, depression and anxiety).^4,5^ Various literatures state the prevalence of internet addiction ranging from 7.4% to 46.4% among students.^6^ Recent study from Nepal reported 51.2% of the respondents had moderate to severe level of internet addiction.^7^

Studies on internet addiction among students of government school are limited in Nepal and no study has been done in this part of Nepal. Considering its high prevalence^7^ and consequence of the problem, it is essential to determine the prevalence of internet addiction among school going students in Rupandehi, Nepal.

The study was conducted with to find out the prevalence of internet addiction among students of government school of Rupandehi, Nepal.

## METHODS

An observational cross-section study was conducted among school going students of class 10 of Shanti Model Secondary School, Tilottama, Rupandehi, Nepal. Ethical approval was obtained from the Institutional Review Committee of same institute (Reference number: 18/2023). Data was collected from October 2023 to November 2023.

Those students who present in school at the time of data collection and gave consent for the study were included in the study. Students with intellectual disability, who did not give consent, and those who were absent during the day of data collection were excluded from our study. A convenience sampling method was used. The sample size was determined based on a 95% Confidence Interval (CI) with a prevalence of 51.2% from a recent study and a 5% margin of error.^7^ The initial calculation yielded a minimum required sample size of 384. To account for a 20% non-response rate, the final sample size was set at 461.

A self-designed structured proforma was devised to obtain the socio-demographic characteristics of the study population. A standard tool Young Internet Addiction Test (IAT) developed by Young was used to find out if addiction was present or not. This tool was freely available in internet. The scale consists of 20 items rated on a 5-point Likert scale yielding a total score categorizing addictive behavior into four categories: no addiction (0-30), mild signs of addiction (31-49), moderate signs of addiction (50-79), and severe addictive behavior (80-100).^3^

Data was collected in school during school hours after getting data collection permission from the school principal. Eligible participants were explained about the purpose of the study, confidentiality assured, and were informed that they could withdraw from study. Verbal informed consent was taken from them. The questionnaire was distributed to students in the classroom and returned back in the presence of researcher. Questions were clearly read out and explained by the researcher to make it clear and easy for the respondents.

Data were entered and analyzed using Statistical Package for the Social Sciences version 24.0. The Point estimate at 95% Confidence Interval was calculated along with frequency and percentages for binary data, and mean and standard deviation for continuous data.

## RESULTS

Among 461, mild to moderate internet addiction was present in 158 (34.27%) of students. There were 226 (49.02%) male and 235 (50.98%) female. The mean age of students was 14.93±0.73 years (Table 1).

**Table 1:** Sociodemographic profile of the participants (n=461).

**Characteristics of the respondents**		Frequency (%)
Mean age (years) ± SD		14.93±0.73
Gender	Male	226 (49.02)
	Female	235 (50.98)
Fathers education	Illiterate	11 (2.39)
	Primary	91 (19.74)
	Secondary	225 (48.81)
	Higher secondary	91 (19.74)
	Masters	43 (9.33)
Mothers education	Illiterate	25 (5.42)
	Primary	103 (22.34)
	Secondary	226 (49.02)
	Higher secondary	74 (16.05)
	Masters	33 (7.16)

SD: Standard Deviation

A total of 388 (84.16%) students used mobile phones for internet access, and 255 (55.31%) reported using the internet primarily for entertainment. YouTube was accessed by 286 (62.04%) students, TikTok by 77 (16.70%), and Facebook by 46 (9.98%). A total of 252 (54.66%) respondents used the internet for less than one hour per day, while 55 (11.93%) spent more than two hours online. A total of 399 (86.55%) students accessed the internet at home (Table 2).

**Table 2:** Internet use pattern among participants (n=461).

**Characterstics of internet use pattern**		Frequency (%)
Device used for internet	Mobile	388 (84.16)
	Laptop/Desktop	50 (10.85)
	Ipad/tablet	16 (3.47)
Major purpose of internet use	Entertainment	255 (55.31)
	Academic	206 (44.69)
Most commonly viewed in internet	Youtube	286 (62.04)
	Ticktok	77 (16.70)
	Facebook	46 (9.98)
	Instagram	26 (5.64)
	Games	26 (5.64)
Duration of internet use per day	<1 hour	252 (54.66)
	1 hrs to 2 hrs	154 (33.41)
	2hrs-3hrs	55 (11.93)
Place of internet use	Home	399 (86.55)
	School	7 (1.52)
	Both	55 (11.93)

**Table 3:** Grading of internet addiction (n=461).

Level of Addiction	n (%)
No addiction	303(65.73)
Mild addiction	137(29.72)
Moderate addiction	21(4.56)
Severe addiction	-

Out of total Students 137 (29.72%) had mild internet addiction, 21 (4.56%) had moderate internet addiction and 303 (65.73%) had no internet addiction (Table 3).

**Table 4:** Comparison of socio-demographic variables and internet use pattern among internet addiction respondents (n=461).

Variable	Internet addiction Present n (%)	Internet addiction Absent n (%)
**Gender**		
Male	78 (34.51)	148 (65.48)
Female	80 (34.04)	155 (65.95)
**Education of father**		
Illiterate	4 (36.36)	7 (63.64)
Basic education (below SLC)	113 (35.75)	203 (64.24)
Higher education	41 (30.59)	93 (69.40)
**Education of mother**		
Illiterate	9 (36.00)	16 (64.00)
Basic education (below SLC)	116 (34.21)	213 (65.78)
Higher education	33 (30.84)	74 (69.15)
Purpose of internet use		
Academic	53 (25.72)	153 (74.27)
Entertainment	105 (41.18)	150 (58.82)
**Most frequently viewed**		
YouTube	72 (25.17)	214 (74.82)
Others	86 (49.14)	89 (50.85)
Most common place to use internet		
Home	132 (33.08)	267 (66.91)
School	2 (28.57)	5 (71.42)
Both	24 (43.63)	31 (56.36)
**Duration of internet use**		
More than 2 hours	32 (58.18)	23 (41.81)
Less than 2 hours	126 (31.03)	280 (68.96)
**Type of gadget used**		
Mobile	123 (31.70)	265 (68.29)
Others	35 (47.94)	38 (52.05)

## DISCUSSION

Our study showed that almost one third 34.6% of the students had mild to moderate internet addiction which is lower than the study conducted at Gandaki Province, Nepal which reported more than half 51.2% having internet addiction.^7^ However this study was conducted at private school only. The reason for low prevalence in our study might be because we included students of only class 10 where students spend most of their time in school because of upcoming SEE exam. Another reason might be difficult accessibility of internet at school/and mobiles which are the easy means of internet access were not allowed in the school premises. Studies has shown that adolescent from private schools have higher level of internet addiction when compared with Government school students.^8^ In the study conducted in Pokhara7 students of both class nine and ten were included in the study. Another study conducted in Western Nepal reported prevalence of internet addiction among school going adolescent to be 73.5%. This study found that 64.7% of adolescents had mild internet addiction and 8.8% had moderate addiction.^9^

Our study had almost similar finding with the studies conducted in different parts of India.(10-12) Studies from Middle East country also showed similar findings which reported 29.6% school students having moderate to severe internet addiction.^13^

However, some Indian studies contrasts with our finding where prevalence of internet addiction among school students were found to be 11.8%.^14^ The variations in prevalence of Internet addiction across different country or even in the study conducted in same country might be because of sample size, sampling procedure, difference in social context and background of the participants, purpose of the Internet use, tools used and Internet Addiction scoring cut-off points used in same tools in the studies.

The study showed majority of the respondent 83.7% were using internet in mobile which is consistent with findings from other Nepalese studies.^7,9^ The respondents were using internet in mobile because of easy accessibility of mobile phones and easy access to internet package on mobile phone.

We detected slightly higher prevalence in male students 78 (34.51%) than in female students 80(34.04%) in this setting, which is similar to studies conducted in India and abroad.^12,14^ It may be explained by the fact that males are more attracted to the wider utility of the internet such as online games than females.^15^ Several studies have indicated that gender is one of the predicting factors in Internet addiction, that is, males are more likely than females to become Internet addicts.^16^

Prevalence of Internet addiction in our study was found to be higher among those who spent more time in internet 32 (58.18%). This finding is similar with the study conducted in India which reported students with internet addiction spent longer time online as compared to non addicts with an average of around 18 hours/week.^17^

Majority of the participants 255 (55.30%) used internet for entertainment purpose followed by academic purpose. This finding is consistent with the study conducted in Nepal where 65.3% students were using internet for recreation purpose.^7^ In a study conducted in undergraduates students in Kathmandu majority of the participants had used internet for entertainment and refreshment purpose followed by education or to get new information.^18^

Facebook and YouTube were among most visited web-sites which is consistent with other similar studies.^17,19,20^

The limitation of our study was that it was conducted in single government school of Rupandehi district. Due to this, the generalizability of this study finding may not represent to all other school going students. So, further studies using larger sample size is necessary.

## CONCLUSIONS

This study highlights that approximately one-third of students in a government school in Rupandehi, Nepal, exhibit mild to moderate levels of internet addiction. The findings suggest that internet use is predominantly for entertainment, with mobile phones being the most common device used. While the prevalence of internet addiction is lower in this study compared to some previous research in Nepal.
